# The Impact of Health Insurance Programs on Out-of-Pocket Expenditures in Indonesia: An Increase or a Decrease?

**DOI:** 10.3390/ijerph10072995

**Published:** 2013-07-18

**Authors:** Budi Aji, Manuela De Allegri, Aurelia Souares, Rainer Sauerborn

**Affiliations:** 1 Institute of Public Health, Faculty of Medicine, University of Heidelberg, Im Neuenheimer Feld 324, Heidelberg 69120, Germany; E-Mails: Manuela.De.Allegri@urz.uni-heidelberg.de (M.D.A.); souares@uni-heidelberg.de (A.S.); rainer.sauerborn@urz.uni-heidelberg.de (R.S.); 2 School of Public Health, Faculty of Medicine and Health Sciences, Jenderal Soedirman University, Kampus Karangwangkal, Purwokerto 53123, Indonesia

**Keywords:** health insurance, out-of-pocket expenditures, panel data analysis

## Abstract

We used panel data from the Indonesian Family Life Survey to investigate the impact of health insurance programs on reducing out-of-pocket expenditures. We employed three linear panel data models, two of which accounted for endogeneity: pooled ordinary least squares (OLS), pooled two-stage least squares (2SLS) for instrumental variable (IV), and fixed effects (FE). The study revealed that two health insurance programs had a significantly negative impact on out-of-pocket expenditures by using IV estimates. In the IV model, *Askeskin* decreased out-of-pocket expenditures by 34% and *Askes* by 55% compared with non-*Askeskin* and non-*Askes*, respectively, while *Jamsostek* was found to bear a nonsignificant effect on out-of-pocket expenditures. In the FE model, only *Askeskin* had a significant negative effect with an 11% reduction on out-of-pocket expenditures. This study showed that two large existing health insurance programs in Indonesia, *Askeskin* and *Askes*, effectively reduced household out-of-pocket expenditures. The ability of programs to offer financial protection by reducing out-of-pocket expenditures is likely to be a direct function of their benefits package and co-payment policies.

## 1. Introduction

In the past two decades, several Asian countries, including Indonesia, have implemented social health insurance in the process of restructuring their health care financing systems and improving access to health services by reducing the price at point of use for medical services. Most studies from these developing countries found that health insurance had a positive impact on increasing health care utilization [1,2,3,4]. However, evidence on the adequacy of social health insurance in providing financial protection, by alleviating the burden of out-of-pocket spending, still appears contradictory. For example, the existing health insurance systems in China and India have shown limited success in reducing financial risk or in protecting households from catastrophic health spending [5,6]. Conversely, health insurance for the poor in Vietnam did reduce out-of-pocket spending [7,8]. One of the reasons for the conflicting evidence is the differences in the health insurance structures and the contexts in which they operate. This makes it necessary to investigate and evaluate insurance systems for their performance with respect to their targeted impacts in the pertinent settings [9]. To date, the health insurance and out-of-pocket payments nexus has become central to the debate on effective health care financing mechanisms [3,5,7,8].

In Indonesia, the health care financing system has been set up to include a mix of public and private financing, of which the latter still plays a dominant role. To a large extent, this has led to a fragmented and segmented health insurance structure. After the implementation of the Social Security Act in early 2005, Indonesia has three large health insurance schemes that differ in population coverage, benefits packages, and insurance agencies: namely *Askes*—for civil servants (introduced in 1968); *Jamsostek*—for private formal employees (introduced in 1992); and *Askeskin*—for poor people (introduced in 2005). This substantial development of the health insurance market obviously influenced subsequent demand for health services. Hidayat* et al.* [10] confirmed that both *Askes* and *Jamsostek* had a strong positive impact on increasing demand for outpatient care. Furthermore, the World Bank reported that after the implementation of *Askeskin*, the utilization rates for outpatient and inpatient care increased by nearly 50% [11]. These results are consistent with the argument that health insurance increases the demand for health services by reducing the costs at point of use. This phenomenon is known as “ex post moral hazard” [12].

The increasing demand for health services due to insurance raises a question as to whether it is also followed by increasing financial protection against the cost of illness among insured people. The purpose of health insurance is not only to tear down the barriers to the access of health services due to financial reasons, but also to ensure further financial protection [13]. Theoretically, health insurance should prevent excessive out-of-pocket expenditures. However, we postulate that supplier-induced demand may subvert the financial protection offered in principle by health insurance. This may occur as people who would have not reached the facilities in the absence of insurance are now encouraged by providers to use several treatments and procedures, still subject to copayments. This increased utilization and its link to existing supplier-induced demand may actually result in higher out-of-pocket expenditures and hence in an increased financial burden for those with a valid insurance [14]. Health insurance in Indonesia is potentially vulnerable to this supplier-induced demand, as by its own design, it lacks the infrastructure for utilization review,* i.e.*, a mechanism to monitor what services and in what quantities are prescribed.

From a methodological prospective, a crucial aspect when assessing the effect of health insurance on health care expenditures is the problem of endogeneity [3,5,15,16]. Endogeneity refers to the fact that an explanatory variable is correlated with unobservables, relegated to the error term. Measurements of the impact of insurance on out-of-pocket expenditures without controlling for the problem of endogeneity may be misleading [5]. Several studies to date have adopted different econometric approaches to deal with these issues. Wagstaff [8] used difference-in-difference to measure the impact of the health care fund for poor people in Vietnam, and found that this program reduced out-of-pocket spending. Shaefer* et al.* [17] used an instrumental variable (IV) approach to examine the effect of transitions from private to public health insurance by children on out-of-pocket and insurance premium costs in the U.S. and found that these transitions offered a cash-equivalent transfer of nearly US$1,500 annually in the form of reduced spending. Moreover, Sepehri* et al.* [18] concluded that failure to capture endogeneity resulted in different study outcomes against the basic argument that health insurance eliminates excessive household financial expenditures on health care.

In view of the available evidence, this study aimed to investigate the effects of various health insurance schemes in Indonesia on reducing out-of-pocket expenditures, correcting for underlying endogeneity. We used panel household survey data gathered at four points in time points 1993 and 2007.

## 2. Health Insurance in Indonesia—Sources of Endogeneity

With approximately 237 million people in 2010, Indonesia is the 4th largest country in the World [19]. Its population is spread over five major islands and 30 small groups of islands, covering more than 17,000 individual islands. National public health expenditure has been rising gradually from 2001 to 2006, but the levels still remain below 1% of Gross Domestic Product (GDP) [20].

Table 1 provides summary characteristics of the three available health insurance schemes in relation to payment systems, health care provider network, and services covered [10,21]. Efforts to scale up health insurance in Indonesia have evolved substantially since the drafting of Law No. 40 in 2004, that ratified the National Social Security System. This law marked governmental commitment to reform the existing social protection system to advance universal coverage by promoting nation-wide social health insurance. By the first quarter in 2005, the government had already achieved remarkable progress in providing health insurance for the poor and vulnerable groups (*Askeskin*), funded through the public budget [21,22]. This program has gradually increased the number of insured people in Indonesia. A target of 36.1 million (17% of the total population) covered individuals was set for the first semester of 2005 and a higher target of 60 million for the second semester of the same year. By mid-2007, *Askeskin* was estimated to cover 76.4 million people [23].

The implementation of *Askeskin* caused nation-wide health insurance coverage to increase from 10% in 2005 to 48% of the total population in 2008 [21]. *Askeskin* includes non-contributory premiums and no cost sharing for all health benefits. However, this scheme does not include treatments categorized as luxury treatments.

Target *Askeskin* beneficiaries were identified on the basis of the poverty listing drawn by the Indonesian Bureau of Statistics. The national government allocated fixed quotas to districts, and districts had responsibility to identify the target beneficiaries. Targeting proved challenging, so that during the initial enrollment phase, a number of shortcomings was actually highlighted in *Askeskin* program. A rapid assessment report conducted by Bachtiar* et al.* [11] points out that *Askeskin* was sometimes allocated based on health status. Endogeneity in estimating the impact of *Askeskin* on financial protection may arise as sicker people might have self-selected into the scheme to benefit from its generous benefits package.

**Table 1:** Main features of the three health insurance schemes in Indonesia.

Characteristics	Health Insurance Schemes		
	*Askes*	*Jamsostek*	*Askeskin*
Established	1968	1992	2005
Population coverage	14 million (about 6% of the population) in 2007	4.1 million (about 2% of the population) in 2009	76.4 million (about 34% of the population) in 2007
Participation	Mandatory	Mandatory, opt-out option for employers that could provide better benefit plans	Social insurance
Organization/Carrier	State-owned company (*PT ASKES* Indonesia)	State-owned company (*PT JAMSOSTEK* Indonesia)	Ministry of Health
Beneficiaries	Civil servants, pensioners of civil servants and armed forces	Formal private employee	Identified poor and near poor, based on individual and household targeting
Eligible dependents	Spouse and 2 oldest children <21 years of age (if unemployed, unmarried), or <25 years of age if a full-time student	Spouse and 3 oldest children <21 years of age	Spouse and children
Source of funds	Member contribution 2% of basic salary + contribution from government 2% of basic salary	Member contribution, if single: 3% of basic salary; member with dependents: 6% of basic salary	No contribution from beneficiaries because it is tax-based with calculation for premium 6,000 IDR (0.46€) *per capita*
Benefits package and provider choice	Outpatient and inpatient care at public providers only	Outpatient care at both public and private providers networks, and for inpatient care at public providers only	Outpatient and inpatient care at public providers only
Negative list of benefits package	Cosmetic surgery, physical check-up, alternative medicine, dental prostheses, fertility treatment, non-basic immunization	General check-up, cancer treatment, heart surgery, renal dialysis, and lifelong treatment for congenital diseases, prostheses, non-basic immunization, transplantation, fertility treatment	Cosmetic surgery, physical check-ups, alternative medicine, dental prostheses, fertility treatment
Copayment	Yes, if members want to upgrade class, branded drugs out of formulary, renal dialysis, transplantation, heart surgery	None, but *Jamsostek* does not cover high cost treatments such as cancer treatment, heart surgery, and renal dialysis	None
Provider payment arrangement	Primary care: capitation Secondary care: fee schedule	Primary care: capitation Secondary care: capitation and fee schedule	Primary care: capitation Secondary care: negotiated fee with limit

Source: Abstracted from Hidayat* et al.* and Roxt* et al.* [10,21]**.**

*Askes* is a contributory social insurance (for civil servants, pensioned civil servants and armed forces with their dependents). This scheme is managed by the state-owned enterprise, *PT Askes*, and covers about 14 million people. Member contribution is set at 2 percent of monthly base salary, with the government contributing an additional 0.5% since 2003. This scheme provides comprehensive benefits for both outpatient and inpatient care through a structured health provider mechanism. A cost sharing policy is applied for certain medical treatments [10,21,24].

Endogeneity in estimating the impact of *Askes* on financial protection may arise, as enrollment is dependent on social servant status. *Askes* provides comprehensive benefits packages upon retirement, so this may be a choice variable for individuals in Indonesia. Poorer health status individuals, in anticipation of their high future medical needs, may be more likely to choose to be civil servants, knowing that such a position grants them access to health insurance. Healthier individuals may be more likely to seek a job in an enterprise or to choose self-employment, knowing no portion of their earnings will be deducted for health insurance [25].

*Jamsostek* is a social insurance fund targeting workers in the formal economy, specifically in firms with at least 10 employees and with a minimum wage of 1 million IDR (80.50€) per month. The insurance premium is fully the employer’s responsibility and is set at 3% of the monthly base salary for single workers and at 6% for workers with dependents. The scheme covers about 4.1 million employees and their dependents. *Jamsostek* provides a comprehensive benefits package and allows the members to access both public and private outpatient provider networks, but inpatient care is limited to public hospitals. However, this scheme does not cover catastrophic health care. Participation into *Jamsostek* is relatively low due to an opt-out provision for employers if they can provide better private insurance coverage than the *Jamsostek* benefits package. *Jamsostek* only covers approximately 7% of total formal sector employees. The participation of the employers into *Jamsostek* is subject to option, and employers choosing to select out of the program if they have other options. This opt-out clause or optional membership policy has resulted in adverse selection for *Jamsostek*. Some employers purchase a commercial insurance plan for their employees, and many employers still provide no protection for their employees [21,23,26,27,28].

## 3. Methodology

### 3.1. Data

The study was based on data from the Indonesia Family Life Survey (IFLS) dataset. The IFLS dataset consists of panel household data stretched over four time periods. The sample represents 83 percent of the population in 13 out of the 27 Indonesian provinces and is stratified to include various cultural and socioeconomic backgrounds. The IFLS was first conducted in 1993, covering 7,224 households. Additional rounds were conducted in 1997, 2000, and 2007. 87.6% of all initially sampled households were included in all four rounds. For a detailed description of the IFLS, see Frankenberg and Thomas and Strauss* et al.* [29,30,31].

The IFLS gathers information on socioeconomic and health seeking behavior at both the individual and at the household level. Across rounds, interviews were conducted in the national language (*Bahasa*), however, interviewers sometimes also mixed in the local language to facilitate the interview process. The econometric model was developed and tested empirically by using panel data from the four rounds of the IFLS. Table 2 presents descriptive statistics for the main variables.

### 3.2. Outcome Variable

Out-of-pocket expenditure was measured as the amount of household expenditure paid as a consequence of obtaining health care during the previous one year. Health expenditure was consistently reported in all four IFLS rounds (1993, 1997, 2000 and 2007). We defined out-of-pocket expenditures to include: hospitalization costs, clinic charges, physician fees, traditional healer fees, and medicines. Expenditure incurred on transportation to access health services was not included in the analysis, because it was not available in the dataset. To remove the effect of inflation, we adjusted annual expenditure values using the Consumer Price Index (CPI) to 2007 values [32]. We set the unit of analysis at the household level. We adjusted for household size and age structure, converting aggregated household values into *per capita* expenditure [33].

**Table 2:** Descriptive statistics IFLS household panel 1993–2007.

Variable	1993		1997		2000		2007	
	N = 7,194		N = 6,667		N = 6,703		N = 6,335	
	Mean	Std. Dev.	Mean	Std. Dev.	Mean	Std. Dev.	Mean	Std. Dev.
Out-of-pocket expenditures (IDR)	167,072.80	806,423.10	182,327.30	816,080.70	171,308.30	2,033,662.00	186,134.00	843,351.20
Household income (IDR)	5,310,577.00	5,310,577.00	6,999,397.00	1.22e+07	6,447,424.00	7,573,455.00	7,862,883.00	2.48e+07
Health insurance								
*Askes*	0.086	0.281	0.147	0.354	0.134	0.340	0.129	0.335
*Jamsostek*	0.010	0.097	0.070	0.255	0.067	0.251	0.074	0.262
*Askeskin*	-	-	-	-	-	-	0.179	0.383
Male	0.839	0.368	0.822	0.382	0.816	0.387	0.781	0.414
Married	0.368	0.377	0.815	0.389	0.805	0.396	0.765	0.424
Education:								
Below junior high	0.687	0.464	0.691	0.462	0.669	0.471	0.638	0.481
Junior high	0.118	0.322	0.114	0.318	0.124	0.329	0.124	0.328
Senior high	0.139	0.346	0.140	0.347	0.143	0.350	0.163	0.369
University	0.056	0.231	0.055	0.227	0.062	0.241	0.077	0.266
Household size >4	0.471	0.499	0.594	0.491	0.674	0.469	0.756	0.429
Age composition (years):								
0–5	0.115	0.151	0.088	0.124	0.074	0.106	0.048	0.084
6–17	0.245	0.220	0.243	0.197	0.231	0.186	0.175	0.160
18–59	0.545	0.245	0.564	0.230	0.584	0.226	0.662	0.211
60 and above	0.095	0.220	0.105	0.216	0.111	0.212	0.115	0.194
Urban	0.475	0.499	0.452	0.498	0.455	0.498	0.493	0.500
Ethnicity (Javanese)	0.587	0.492	0.589	0.492	0.590	0.492	0.585	0.493
Health status								
GHS is “poor”	0.200	0.400	0.270	0.444	0.325	0.468	0.356	0.479
ADL with limitation	0.297	0.457	0.535	0.499	0.627	0.484	0.595	0.491

### 3.3. Explanatory Variables

#### 3.3.1. Income

We used household consumption as a proxy of income given that in low and middle income settings, consumption has been identified to be a more reliable measure of living standard than income [10,33]. We adjusted annual consumption values using the CPI to the 2007 value. *Per capita* consumption levels were computed by adjusting household values for household size. This method of computing socio-economic status allows accounting for intra-household spillover effects,* i.e.*, intra-household resource transfer or cross subsidies [33].

#### 3.3.2. Health Insurance

We analyzed the three largest health insurance schemes implemented in Indonesia: civil servant health insurance (*Askes*), employee health insurance (*Jamsostek*), and health insurance for poor people (*Askeskin*). The survey asked whether the head of the household was enrolled in one of these insurance schemes which also automatically included household dependents. The insurance types were included in the model as dummy variables.

#### 3.3.3. Health Status

Health status is one of the determinants driving health seeking behavior. Sick people are more likely to seek health care services from traditional or modern (either public or private) providers than healthier people. Ha* et al.* [34] found that households with sick people were correlated with choosing health care providers. Gotsadze* et al.* [35] confirmed that perceived seriousness of illness was a significant factor which increased the probability to seek for health care. Patients with high levels of illness perception were more likely to seek health care than those with low level of perception, which suggests that patients with higher perceived illness would spend more or have higher medical expenditures than those with lesser degree of perceived illness.

We included two indicators of health status in the models: self-rated general health status (GHS) and activity of daily living (ADL). These are two measures of self-reported illness that reflect the need for health care and derived from individuals’ responses to the health-related questions in the IFLS surveys. The GHS measures were based on individuals’ assessment of subjective health status. The questions on the GHS asked individuals to rate their general health status on a 4-point categorical scale: very good, good, bad and very bad. For the purpose of this study, we aggregated very good and good into one response category, and bad and very bad into another one. A dummy indicating whether members in the household had a “poor” health status was included. The ADL measures were based on individuals’ self-ratings of ability to perform the basic tasks of everyday life, such as carrying a heavy object 20 meters, climbing stairs, walking, bending, kneeling or stooping, drawing water from a well, dressing without assistance, rising from a sitting position in a chair, toileting, and rising from a sitting position on the floor. A dummy indicating whether household members had difficulty or an inability to perform these activities was also included.

#### 3.3.4. Age Composition of Household

Because the unit analysis of this study was the household, individual age could not be used in this model. As an alternative, we computed the percentage of the household in different age groups. This grouping reflected patterns of morbidity associated with different ages: (a) <6 years; (b) 6–17 years; (c) 18–60 years; and (d) >60 years. 

#### 3.3.5. Other Variables

Other independent variables included demographic variables such as married (a dummy indicating household head is currently married), male (a dummy indicating household head is male), family members (a dummy indicating family members are more than four persons), urban (a dummy indicating residence in urban area), education of the household head (dummies indicating below junior high (reference), senior high, and college and university), and ethnicity (a dummy indicating Javanese).

### 3.4. Econometric Model

We used three different econometrics approaches in the selection process of an appropriate model based on the principle of panel data analysis, the sampling design of study, and the nature of health insurance uptake. *Askes*, *Jamsostek* and *Askeskin* have different dates of establishment, enrollment mechanisms, benefits schemes, and population targets. To address these issues, we conducted an econometric identification strategy that allowed for robust and unbiased estimation and also facilitated the interpretations of the parameters. All estimation was carried out using Stata version 12.1.

To investigate the effects of health insurance on out-of-pocket payments, we first employed a pooled ordinary least squares (OLS) model covering the whole period, 1993–2007. We constructed a pooled OLS regression as follows:

ln *oop_it_* = *β*_0_ + *β*_1_*x_it_* + *β*_2_*Askes_it_* + *β*_3_*Jamsostek_it_* + *β*_4_*Askeskin_it_* + *β*_5_*z_it_* + *β*_6_*y_t_* + *α_i_* + *ε_it_* (1)

where *i* = 1,...,n represents households and t = 1993, 1997, 2000, 2007 represents years. *oop_it_* is *per capita* out-of-pocket expenditures for household *i* at period *t*. To reduce the effects of the skewed nature of the out-of-pocket expenditures variable in the equation, the dependent variable (*oop_it_*) was log-transformed. The treatment variable was defined as: *Askes_it_* = 1 if the household was enrolled in *Askes*, and *Askes_it_* = 0 otherwise; *Jamsostek_it_* = 1 if the household was enrolled in *Jamsostek*, and *Jamsostek_it_* = 0 otherwise; *Askeskin_it_* = 1 if the household was enrolled in *Askeskin*, and *Askeskin_it_* = 0 otherwise. *x_it_* is a vector of time-variant specific effects such as household head sex, marital status, education of the household head, and household size, age composition, location, income, and health status,. *z_it_* denotes a vector of time-invariant household characteristics such as ethnicity. *y_t_* denotes year dummies that capture time shock; *α_i_* is *a* vector of unobserved time-invariant specific effect and *ε_it_* is an idiosyncratic error or time-varying error which was assumed to be randomly distributed.

Note that in Equation (1), the OLS estimates of* β_2_*,* β_3_* and* β_4_* are still heavily biased because of endogeneity, that is, a correlation between insurance status and the error term,* α_i_*. The problem of adverse selection in the schemes creates a higher likelihood of correlation between insurance status and the error term (*α_i_*) which induces a bias in the coefficient of health insurance on the out-of-pocket expenditures equation. This condition leads to a positive association between insurance status and out-of-pocket expenditures because higher health risk people are more likely to enroll in an insurance plan than others. To address this endogeneity problem, we considered two options: to develop an instrumental variable (IV) model estimated on the pooled panel data and a fixed effects (FE) model.

We first addressed endogeneity through the application of an instrumental variable model. The IV on the pooled panel data provides a consistent estimator under the strong assumption that a valid instrument exists, meaning that the instrument is correlated with insurance status and uncorrelated with the error term. As in prior applications of the same [36], the instrument is used to control the error term but it does not lead to a direct change in the outcome variable.

In the IV model, we treated insurance status as an endogenous variable. We tested for possible endogeneity of the regressor using the Hausman test to measure the difference between OLS and IV estimator and the related Durbin-Wu-Hausman (DWH) test to produce a robust test statistic. A significant difference between OLS and IV model estimates, as indicated in the Hausman test, would suggest the exogenous status of insurance. Similarly, a significant DWH test would also suggest that insurance is endogenous [36].

We constructed pooled two-stage least squares (2SLS) to calculate IV estimates for insurance status. We conducted the instrument relevance and validity tests to indicate that our instruments were correlated with insurance status, but uncorrelated with error term (*α_i_*). For the relevance of the instruments, we employed several key diagnostic statistics to identify weak instruments. We evaluated the *R^2^* of the first-stage reduced-form equation and the *F*-test of the joint significance of the instruments excluded from the structural model. Given one of our models had two suspected endogenous regressors (*i.e.*, *Askes* and *Jamsostek* variables), the *R^2^* value and *F* statistic might not have been sufficient to diagnose instrument relevance. Therefore, we also used a Shea partial *R^2^* measure that investigates inter-correlations among instruments. For the validity of the instruments’ diagnostic, we applied an over-identification test by using Sargan statistic. Then, for the original set of orthogonality conditions of the instruments’ testing, we employed *C*-statistic. The *C* test allows to confirm whether selected instruments are exogenous [36,37,38].

We used the eligibility for the government social protection program as an instrument for *Askeskin* variable (*g_it_*). However, given this eligibility criteria was only relevant for the surveys from 2000 and 2007, we limited our analysis related to the *Askeskin* scheme only to these two data collection points. To assess the impact of both *Askes* and *Jamsostek* on household out-of-pocket health expenditure, we employed the same set of three instrumental variables (*e_it_*): household participation in community meetings, household participation in women’s group organizations, and workplace size (workplace with at least 10 employees). The first two instruments were successfully used in a prior study in Indonesia [25]. We assumed that these instruments were correlated with the probability of being insured in either scheme and uncorrelated with out-of-pocket expenditures only through insurance once we had controlled for health insurance and other covariates. For these two schemes, we constructed a model that used only three waves of panel data (*i.e.*, 1997 to 2007), due to the availability of the information needed to construct the instruments.

We controlled for autocorrelation in the error term by applying cluster-robust standard errors. Since instrumental variables are aggregated at the household level, we used household level as clustering option. Furthermore, in case of *Askeskin*, we also considered another form of shock from alternative targeted poverty programs. These programs could lead to confounding effects of *Askeskin*, particularly while there was the overlap of the target groups, targeting criteria and mechanisms [39]. We therefore included subsidized rice variable as a potential confounding factor for *Askeskin* to control some non-trivial overlap between both programs. Subsidized rice program introduced in 1998 under social safety net in response to the 1997 economic crisis. The information of subsidized rice program was also available in the IFLS 2000 and 2007. We constructed a subsidized rice recipient dummy variable in the IV model for *Askeskin* to correct for this issue.

Lastly, a fallback of the IV model was that we could only count on partial observation (*i.e.*, 2000 and 2007 for *Askeskin* scheme and 1997-2007 for *Askes* and *Jamsostek* schemes) leading to rather imperfect estimates. We therefore also addressed the problem of endogeneity through the application of an FE model covering the entire IFLS dataset (*i.e.*, 1993–2007). FE models offer consistent parameter estimates, provided that endogeneity is due to the correlation between unobserved time-invariant specific effects (*α_i_*) and insurance status and that insurance is uncorrelated with *ε_it_*. FE models consistently provide an unbiased estimator of insurance by taking advantage of differencing transformation that eliminates *α_i_* [34] (*i.e.*, health care preference and static socioeconomic characteristics). This model also included the control variable, *x_it_*, for change in gender of household heads, marital status of household heads, education of household heads, household size, age composition, location, and income. Our FE model did not include time-invariant variables, such as ethnicity. In our FE model, we also applied cluster-robust standard errors, assuming that observations are independent over the number of time periods.

## 4. Results

### 4.1. Descriptive Analysis

Table 3 reports the sample distribution and the percentage of insured people in each scheme, across the four survey periods. The sample of this study decreased over the years. From 7,224 original households in IFLS1, after data cleaning, we selected 7,194 households for our analysis. Table 3 indicates that the number of *Askes* beneficiaries ranged from 9 to 14% of the total sample during survey periods; the number of *Jamsostek* beneficiaries increased from 1% in the initial survey period to 7% in the next periods; and the number of *Askeskin* beneficiaries was at 18% in 2007. From 1993 to 1997, the overall percentage of enrolled households increased substantially. Table 3 also confirms that the introduction of *Askeskin* caused a sizeable increase in the number of health insurance beneficiaries in 2007.

**Table 3:** The distribution of health insurance status across year.

Insurance status	1993		1997		2000		2007	
	n	%	n	%	n	%	n	%
*Askes*	622	9.56	981	14.71	895	13.35	817	12.90
*Jamsostek*	69	0.96	467	7.00	451	6.73	470	7.42
*Askeskin*	0	0.00	0	0.00	0	0.00	1,133	17.88
Uninsured	6,503	90.39	5,219	78.28	5,357	79.92	3,915	61.80
Total households	7,194	100.00	6,667	100.00	6,703	100.00	6,335	100.00

**Figure 1:**
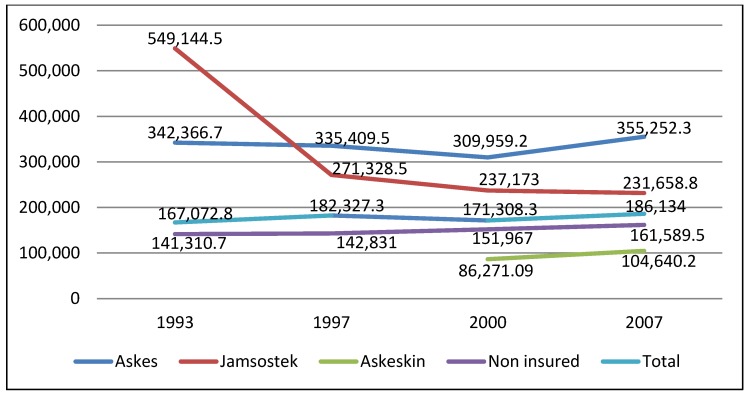
The distribution of mean out-of-pocket expenditures across year in Indonesia Rupiah (IDR) (1€ = 12,421.89 IDR).

**Figure 2:**
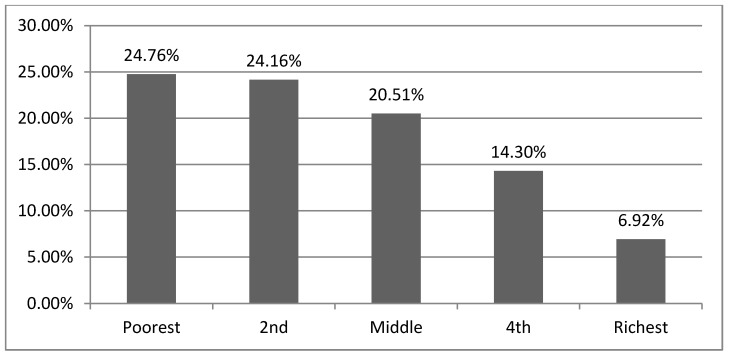
The distribution of *Askeskin* enrollees across different income level (2007).

Figure 1 depicts annual *per capita* out-of-pocket expenditures by insured and uninsured households. *Per capita* out-of-pocket expenditures among *Askes* beneficiaries slightly decreased between 1993 and 2000, but increased by 16% from 2000 to 2007. *Per capita* out-of-pocket expenditures dropped by 50% among *Jamsostek* beneficiaries between 1993 and 1997. A further decrease of less than 1% occurred between 1997 and 2007. Figure 1 also displays *per capita* out-of-pocket expenditures among *Askeskin* beneficiaries before and after the scheme implementation. In 2007, households receiving *Askeskin* benefits spent 20% more than in 2000. This increase might be due to adverse selection, in which non-eligible individuals with high anticipated health expenditures enrolled in the program. As depicted in Figure 2, around 20% of *Askeskin* beneficiaries came from households in higher and highest income quintiles.

### 4.2. Impact Estimation Results

Table 4 reports the results of all three models (simple OLS, IV model, and FE model). We found that the OLS estimates (Models 2 and 4) differed substantially from the IV estimates (Models 3 and 5). The OLS coefficients for *Askes*, *Jamsostek* and *Askeskin*, −0.007, 0.011, and −0.022, respectively, (Models 2 and model 4) differed significantly from the IV coefficients of −0.802 for *Askes* (Model 3), −0.102 for *Jamsostek* (Model 3) and −0.411 for *Askeskin* (Model 5). Model 3 and model 5 produced significant values for *Askes* and *Askeskin*. Both this set of results and the robustified DWH test confirmed the endogeneity of insurance status and led to the rejection of the null hypothesis that *Askes*, *Jamsostek* and *Askeskin* were exogenous (Table 5).

**Table 4:** The effects of health insurance programs on household out-of-pocket expenditures.

Variables	(1) Pooled OLS 1993–2007	(2) Pooled OLS—comparison for (3) 1997–2007	(3) Pooled 2SLS (IV) for *Askes* and *Jamsostek* 1997–2007	(4) Pooled OLS—comparison for (5) 2000–2007	(5) Pooled 2SLS (IV) for *Askeskin* 2000–2007	(6) FE 1993–2007
Health insurance						
*Askes*	0.015 (0.040)	−0.007 (0.045)	−0.802 (0.337) **	-	-	0.008 (0.068)
*Jamsostek*	0.024 (0.051)	0.011 (0.053)	−0.102 (0.261)	-	-	0.017 (0.064)
*Askeskin*	−0.088 (0.055)	-	-	−0.022 (0.059)	−0.411 (0.238) *	−0.111 (0.064) *
*Subsidized rice recipient*	-	-	-	−0.146 (0.036) ***	−0.113 (0.041) ***	-
*Male*	−0.026 (0.049)	0.015 (0.056)	0.004 (0.057)	−0.021 (0.070)	−0.013 (0.070)	0.002 (0.070)
*Married*	−0.049 (0.049)	−0.100 (0.056) *	−0.107 (0.057) *	−0.086 (0.070)	−0.095 (0.070)	0.032 (0.068)
Education: Below junior high *)						
*Junior high*	0.167 (0.037) ***	0.186 (0.042) ***	0.264 (0.055) ***	0.138 (0.051) ***	0.132 (0.051) **	−0.036 (0.062)
*Senior high*	0.209 (0.037) ***	0.237 (0.044) ***	0.431 (0.097) ***	0.171 (0.052) ***	0.156 (0.053) ***	−0.069 (0.073)
*University*	0.273 (0.056) ***	0.273 (0.065) ***	0.661 (0.176) ***	0.196 (0.075) ***	0.179 (0.076) **	−0.201 (0.106) *
*Household size*	−0.073 (0.027) ***	−0.100 (0.032) ***	−0.058 (0.040)	−0.084 (0.041) **	−0.086 (0.041) **	−0.056 (0.046)
Age composition (years): 18–59 *)						
*0–5*	0.699 (0.096) ***	0.757 (0.124) ***	0.648 (0.134) ***	0.864 (0.168) ***	0.890 (0.169) ***	0.975 (0.140) ***
*6–17*	−0.384 (0.067) ***	−0.395 (0.081) ***	−0.450 (0.088) ***	−0.453 (0.102) ***	−0.438 (0.102) ***	−0.124 (0.091)
*60 and above*	0.445 (0.068) ***	0.427 (0.081) ***	0.473 (0.085) ***	0.459 (0.101) ***	0.455 (0.101) ***	0.413 (0.112) ***
*Urban*	0.071 (0.025) ***	0.055 (0.029) *	0.078 (0.034) **	0.043 (0.035)	0.052 (0.036)	0.013 (0.068)
*Ethnicity*	0.193 (0.024) ***	0.246 (0.028) ***	0.255 (0.031) ***	0.210 (0.036) ***	0.203 (0.036) ***	-
Health status						
*GHS is poor*	0.365 (0.025) ***	0.350 (0.028) ***	0.355 (0.028) ***	0.342 (0.034) ***	0.346 (0.034) ***	0.292 (0.029) ***
*ADL with limitation*	0.177 (0.024) ***	0.159 (0.027) ***	0.182 (0.029) ***	0.145 (0.033) ***	0.146 (0.033) ***	0.160 (0.028) ***
Household income: Lowest *)						
*Lower 20%*	0.582 (0.033) ***	0.517 (0.039) ***	0.538 (0.040) ***	0.471 (0.048) ***	0.469 (0.048) ***	0.416 (0.042) ***
*Middle 20%*	1.009 (0.035) ***	0.936 (0.040) ***	0.967 (0.044) ***	0.861 (0.048) ***	0.856 (0.048) ***	0.759 (0.045) ***
*Higher 20%*	1.531 (0.036) ***	1.448 (0.042) ***	1.511 (0.053) ***	1.370 (0.051) ***	1.356 (0.052) ***	1.206 (0.049) ***
*Highest 20%*	2.231 (0.041) ***	2.119 (0.048) ***	2.230 (0.073) ***	1.981 (0.059) ***	1.964 (0.060) ***	1.698 (0.057) ***
Year: 1993 *)						
*1997*	0.024 (0.029)	-	-	-		0.026 (0.031)
*2000*	−0.182 (0.029) ***	−0.204 (0.029) ***	−0.222 (0.030) ***	-		−0.146 (0.034) ***
*2007*	−0.131 (0.034) ***	−0.177 (0.032) ***	−0.203 (0.035) ***	0.070 (0.035) **	0.135 (0.053) **	−0.001 (0.042)
*Constant*	9.282 (0.050) ***	9.388 (0.058) ***	9.380 (0.059) ***	9.356 (0.072) ***	9.350 (0.072) ***	9.595 (0.072) ***
*F-statistic (p-value)*	237.35 ( .00)	179.64 ( .00)	172.48 ( .00)	116.81 ( .00)	116.51 ( .00)	57.85 ( .00)
*R^2^*	0.248	0.233	-	0.219	-	0.095
*No. of observation*	20,168	14,475	14,474	9,140	9,140	20,168
*No. of clusters*	6,969	6,390	6,390	5,883	5,883	6,969

*Note*. Standard errors in parentheses. Standard errors were adjusted for clustering at the household level. *** 1%, ** 5%, and *10% significance levels. *) as reference. OLS, ordinary least squares; 2SLS, two-stage least squares; IV, instrumental variable; FE, fixed effects.

**Table 5:** Endogeneity tests.

Test	*Askes* and *Jamsostek*		*Askeskin*	
	Statistics	*p*-value	Statistics	*p*-value
Durbin-Wu-Hausman chi-square test	χ^2^ = 6.684	0.035	χ^2^ = 2.859	0.091
Wu-Hausman F test	*F* (2,14450) = 3.337	0.037	*F* (1,9118) = 2.859	0.091

In addition, we tested the relevance and validity of the selected instruments based on several key diagnostic statistics. For weak instrument testing (Table 6), the values of the *R^2^* and adjusted −*R^2^* from first-stage regression were around 0.1 to 0.2, showing that there will be considerable loss of precision because of IV estimation. These values were not low enough to indicate a weak-instrument problem. Moreover, the values of Partial *R^2^* and Shea’s partial *R^2^* were similar for both *Askes* and *Jamsostek* insurance, indicating that the instruments were sufficient relevance to explain all the endogenous regressors, and the model was well identified. We also considered *F*-test where the instrument should have an *F*-value greater than 10 to indicate that it is relevant [37,38]. The analysis revealed an *F*-value was 69.775 for *Askes*, 77.467 for *Jamsostek*, and 227.177 for *Askeskin*, which suggests that all instruments were strongly correlated with each insurance status.

**Table 6:** Instruments relevance tests.

Test	*Askes*	*Jamsostek*	*Askeskin*
*R^2^*	0.239	0.100	0.192
Adjusted *R^2^*	0.238	0.099	0.191
Partial *R^2^*	0.019	0.045	0.059
*F*-test	*F* (3,14451) = 69.775 ***	*F* (3,14451) = 77.467 ***	*F* (1,9119) = 227.177 ***
Shea’s partial *R^2^*	0.016	0.038	0.059
Shea’s Adjusted partial *R^2^*	0.015	0.037	0.057

*** significant 1%.

For the validity of the instruments, we conducted an over-identification test to assess whether the instruments were valid instruments (*i.e.*, uncorrelated with error term) and they were correctly excluded from the estimated equation. The value of the Sargan’s statistic tests was 2.236 (*p* = 0.135) for* Askes* and *Jamsostek*, which suggests that all instruments were valid and the models were well specified. Further, for orthogonality condition of the instruments, the value of the *C*-statistic was 2.236 (*p* = 0.135) for* Askes* and *Jamsostek*, which suggests that all instruments were exogenous. For *Askeskin* which was a just-identified model, we did not test exogeneity condition of the instrument. The instrument’s independence from error term can be identified if we have a surfeit of instruments [36]. Based on all specification test findings, we concluded that the selected instruments were appropriate enough to be applied in our model and the IV estimators more robust.

Table 4 shows that *Askes* and *Askeskin* had a significant negative effect on household out-of-pocket expenditures for the IV models (Model 3 and 5), whereas in the FE model (Model 6), only *Askeskin* had a significant negative effect. Otherwise, in all OLS models (Model 1, 2 and 4), all insurances had no significant effect on household out-of-pocket expenditures. According to IV models (Model 3 and 5), two insurances decreased household out-of-pocket expenditures. *Askes* decreased out-of-pocket expenditures by 55% (= *e*^−0.802^ − 1) on average at α = 0.05 and *Askeskin* by 34% (= *e*^−0.411^ − 1) at α = 0.10 compared with non-*Askes* and non-*Askeskin*, respectively. *Jamsostek* was found to have a nonsignificant effect on out-of-pocket expenditures, although the association was negative. Furthermore, according to the FE model (Model 6), *Askeskin* had a significant negative effect on household out-of-pocket expenditures at α = 0.10, that is, *Askeskin* decreased household out-of-pocket expenditures by 11% (= *e*^−0.111^ − 1). However, in this model, *Askes* and *Jamsostek* were found to have nonsignificant effects on household out-of-pocket expenditures.

With regard to other covariates, the four household income dummy variables and the two indicators of health status were found to have a significant positive effect on household out-of-pocket expenditures across all regression models. Age composition of less than 5 years and greater than 60 years had a positive effect, but age composition of 6–17 years had a negative effect in all regression analyses. Conversely, in the FE model, age composition of 16–17 year was found to be nonsignificant. Moreover, in the FE model, household heads with a higher education level, such as a university degree, were found to have a significant negative effect. In contrast, in both OLS and IV models, household heads in every education level (*i.e.*, from junior high school to university) were found to have a significant positive effect.

## 5. Discussion

The results of the three models yielded somewhat contradictory findings. In line with our research question, we focused our discussion exclusively on the impact of health insurance on out-of-pocket expenditures and paid particular attention to the problem of endogeneity, independently from the factors, such as supplier-induced demand or provider moral hazard, which might be at the core of the observed effect. We therefore focused our discussion on the results of IV and FE models that, we believe, address the endogeneity of insurance. The findings of IV model confirmed that that two health insurance programs (*i.e*., *Askes* and* Askeskin*) significantly decreased household out-of-pocket expenditures. Using the IV model lead to identify a substantially different effect of insurance status on out-of-pocket expenditures than the standard OLS model. The IV model produced a robust estimator of the extent to which health insurance programs contributed to reduce household out-of-pocket expenditures, while correcting for possible endogeneity. These results are in line with previously published work by Galarraga* et al.* and Shaefer* et al.* in relation to their evaluation of health insurance in Mexico and in the US, respectively [16,17].

The results of the FE analysis confirmed that only *Askeskin* health insurance significantly decreased out-of-pocket expenditures. In contrast, the FE model did not detect any impact of *Askes* and *Jamsostek* schemes on reducing out-of-pocket expenditures. One possible reason explaining why the model detected a significant effect only for the* Askeskin* scheme may be that the number of households in the sample acting as non-treatment group (*i.e*., non-insured) for *Askes* and *Jamsostek* were highly unlikely to experience entry and exit into the treatment group (*i.e*., the insured group) over the four survey periods, as opposed to *Askeskin* whose “non-treatment” group was defined by a clear time point (2005). This is due to the fact that FE models correct for selectivity by computing differences from mean values [36]. The power to estimate an effect is therefore severely jeopardized if the model does not contain sufficient controls.

Appraising our findings in the light of existing literature is difficult since only a few studies have, to date, investigated the impact of health insurance in Indonesia, with specific focus on out-of-pocket expenditures. Sparrow* et al.* [24] examined the effect of *Askeskin* by using propensity score matching approach and found that the program significantly increased out-of-pocket expenditures particularly in urban areas. Their study, however, was vulnerable to biases since it only conditioned “observables” and ruled out “selection on unobservables” to deal with the endogeneity problem. Our findings on *Askes* cannot be appraised in light of similar studies on Indonesia, but may be compared to findings from Vietnam. Controlling for endogeneity, Sepehri* et al.* [18] found that public health insurance, covering civil servants similarly to *Askes*, reduced out-of-pocket expenditures. Our study also suggested that unlike other programs, *Jamsostek* did not significantly reduce out-of-pocket expenditures. The reason is possibly to be found in its less comprehensive benefits package, not covering high cost medical treatments such as hemodialysis, cancer treatment, cardiac surgery, and congenital diseases. This may lead beneficiaries to experience higher out-of-pocket spending to cover treatment for these conditions. This particular situation may explain why *Jamsostek* members end up paying more for uncovered drugs and tests.

Differences in benefits packages may in fact be at the core of the difference in the observed effects across insurance schemes. *Askeskin* has the most generous benefits package, covering almost all types of care with no cost sharing policy and limited service limitations. This is likely to explain why its observed protective effect on out-of-pocket expenditures was more pronounced than that of any other scheme. *Askes* provides a less generous benefits package than *Askeskin*, although more generous than *Jamsostek*, covering several high cost treatments, but applying cost-sharing. This is likely to explain why its protective effect on out-of-pocket expenditures was less pronounced than *Askeskin*, but still more pronounced than *Jamsostek*.

Our study provides relevant evidence for policy, by establishing that at least two of the existing health insurance programs in Indonesia effectively provide households the needed financial protection against the cost of illness. This is an important starting point in the discussions on how to continue and expand health care financing options in the light of moving towards universal health coverage [21]. This study has confirmed that two health insurances (*i.e*., *Askeskin* and *Askes*), which are the largest insurance scheme in Indonesia, have had a positive impact on the financial protection of its members. Our results can conceivably be extended to other populations for which universal health insurance is policy option. A common argument has been that universal coverage health insurance can lead to better financial protection outcomes in some Asian countries [40,41].

There are several limitations to our study. First, our measure of households’ out-of-pocket expenditures included expenditure on traditional medications, a product not covered by any insurance scheme under analysis. In provinces where traditional medical practices are the preferred health seeking choice, due to long-standing socio-cultural norms, the inclusion of traditional treatments in the measure of out-of-pocket expenditures may inflate the relevant value due to factors which are beyond the control or responsibility of the single insurance schemes. Second, the IFLS data did not include information on transportation costs,* i.e.*, out-of-pocket expenditure that allowed households to reach to the selected health care facility. Many studies revealed that even though treatment is free or covered by the public insurance, transport costs can be so burdensome to actually hamper access to services and/or lead to substantially higher out-of-pocket values than when treatment costs alone are considered [42,43]. Third, our study focused exclusively on the effect of health insurance on actual out-of-pocket expenditures and did not analyze other more comprehensive indicators of financial protection, such as catastrophic and impoverishing health spending. Such an analysis should definitely constitute the object of future research. Fourth, our study could not take into account endogeneity deriving from the fact that expenditure could only be observed for the sub-sample of respondents who decided to utilize services in the first place. Fifth, in case of *Askeskin*, the time intervals between the IFLS panel waves are large, allowing confounding time variant unobservables. Other alternative targeted poverty programs, such as unconditional cash transfers and scholarship programs could lead to confounding effects of *Askeskin*.

## 6. Conclusions

Most of the evidence from our models pointed at the fact that two existing health insurance programs, *Askeskin* and *Askes*, in Indonesia significantly reduced household out-of-pocket expenditures after correcting for endogeneity (selection on unobservables). One scheme, *Jamsostek,* produced no significant impact on the reduction of household out-of-pocket expenditures. The ability of schemes to offer financial protection by reducing out-of-pocket expenditures is likely to be a direct function of their benefits package and co-payment policies. Future research needs to expand on our work to explore the impact of health insurance on more comprehensive financial protection indicators, such as catastrophic and impoverishing health spending.
